# Editorial: Using neurophysiological signals that reflect cognitive or affective state

**DOI:** 10.3389/fnins.2015.00193

**Published:** 2015-05-29

**Authors:** Jan B. F. van Erp, Anne-Marie Brouwer, Thorsten O. Zander

**Affiliations:** ^1^TNO Human FactorsSoesterberg, Netherlands; ^2^Human Media Interaction, University of TwenteEnschede, Netherlands; ^3^Team PhyPA, Technical University BerlinBerlin, Germany

**Keywords:** neurophysiology, cognitive state, mental state, affective state, EEG, Brain-Computer Interface, psychophysiology, physiological computing

## Introduction

The central question of this Frontiers Research Topic is: What can we learn from brain and other physiological signals about an individual's cognitive and affective state and how can we use this information? This question reflects three important issues which are addressed by the 22 articles in this volume: (1) the combination of central and peripheral neurophysiological measures; (2) the diversity of cognitive and affective processes reflected by these measures; and (3) how to apply these measures in real world applications.

## Neurophysiological measures and real world applications

Let us first look at the last issue as it is an important driver of the current research and dictates choices related to the first two, for instance when a specific application requires sensor technology to be portable and easy to set up with little calibration time (Gerjets et al., 2014; Huang et al., 2014; Estepp and Christensen, 2015). The authors of this Research Topic describe a wide variety of applications. Many studies follow a so-called passive Brain-Computer Interface approach (Zander and Kothe, 2011): assessing typically covert aspects of the user state without interfering with the task the user is doing. Applications addressed in this volume include improved learning and training environments (Gerjets et al., 2014; Stikic et al., 2014), adaptive vehicle interfaces (Dijksterhuis et al., 2013; Touryan et al., 2014; Wang et al., 2014), classifying concealed information (Farwell et al., 2014), identifying sleep stages (Huang et al., 2014), and non-verbal communication for patients (Kashihara, 2014). Making these applications viable is not trivial. It requires experiments outside a well-controlled lab environment as initiated in several studies here (Dijksterhuis et al., 2013; Betella et al., 2014; Huang et al., 2014; Lin et al., 2014; Stuiver and Mulder, 2014) and knowledge and technological advancements in the area of classification algorithms that require little training data, are robust against environmental and physiological artifacts in the recorded signal, are able to work in real-time and on single trial data, and generalize over tasks. Unlike a lab experiment where participants are usually instructed to sit still and to perform one single task, users in real world situations will usually be in motion, subject to changing cognitive load or emotional state, and/or be multitasking (Van Erp et al., 2012). Several authors focus on developing methods able to cope with these added complexities in life-like situations. For example, Betella et al. (2014) assesses the reliability of psychophysiological signals reflecting emotional state when people are walking and gesticulating. Mühl et al. (2014) look into the effect of emotional state on workload measures, and show that cross-context classifier training is able to compensate for accuracy decline caused by changes in emotional state (especially so for indices in the frequency domain). Touryan et al. (2014) are able to model the time-on-task decrements in performance by analyzing neural activity. Chavarriaga et al. (2014), Vijgh et al. (2014) and Wang et al. (2014) give examples of real time classification. Vijgh et al. (2014) aim to asses stress state and adjust stressor intensity in a gaming environment in real time, Wang et al. (2014) are able to detect the effectiveness of a drowsiness warning system and continuous signatures of fatigue, Chavarriaga et al. (2014) develop single-trial error-related potential recognition in order to improve human-computer interaction, and Estepp and Christensen (2015) present initial proof that electrodes can be removed and replaced between sessions without dramatic loss in performance. Gerjets et al. (2014), Stikic et al. (2014), Stuiver and Mulder (2014) and Touryan et al. (2014) tackle the question about generalizability over tasks. This is an important issue because if the models and algorithms are highly task-specific, it would mean that each application has to start from scratch in choosing the optimal parameters, gathering data and building classification algorithms. Generic classifiers can possibly shorten the calibration time, increasing the usability and applicability. Stuiver and Mulder (2014) investigate and try to grasp differences in the physiological patterns of an ambulance dispatcher task and driving. Stikic et al. (2014) and Touryan et al. (2014) focus on the validity of their algorithms for different tasks: marksmanship and golf (Stikic et al., 2014), and driving and perceptual discrimination (Touryan et al., 2014). It is not surprising that many authors look at ways to further improve signal processing and classification techniques including neural networks (Casson, 2014; Stikic et al., 2014), feature fusion (Putze et al., 2014), and elastic nets (Hogervorst et al., 2014).

## Constructs of interest

The wide range of foreseen applications also illustrate the broadness of the constructs of interest. Workload, stress, and emotion are classic examples, but there is also an increasing interest in the ability to determine what information the brain is processing (Pineda et al., 2013; Lin et al., 2014) and for instance whether this is visual or auditory (Putze et al., 2014), which in turn links to the multitasking challenge of real world applications. Additional contributions to this area come from studies into new paradigms to evoke specific states as discussed by Gerjets et al. (2014). For instance, Vijgh et al. (2014) describe an automated stress induction and control application and Brouwer and Hogervorst (2014) describe an elegant, simple and ethically acceptable way of inducing mental stress that results in physiological responses comparable to those induced by the Trier Social Stress Test.

## Combining central and peripheral neurophysiological measures

The complexity of the constructs to be measured, and the fact that (neuro-) physiological indices only provide indirect access to them forces this field to explore and combine measures from different sources. Quite novel are particular combinations of central and peripheral measures and combinations of different central sensors (more specifically EEG and NIRS). Strait and Scheutz (2014) start with an interesting analysis of what we can and cannot (yet) do with fNIRS and Putze et al. (2014) provide an example that fusing EEG and fNIRS features can increase the accuracy of classification. Stikic et al. (2014) combine ECG and EEG derived measures to correlate physiological changes with training progress, and Hogervorst et al. (2014) determine the workload classification accuracy of EEG, ECG, skin conductance and eye-based measures, alone and in combination. This study contributes to the long-lasting dispute about the best workload measures in favor of EEG-derived indices. More particular, information derived from a single electrode location (Pz) turns out to be an adequate workload predictor. Finally, papers also demonstrate the use of a wide variety of indices from a single source, for instance EEG is analyzed both in the time domain (P3 event related potentials, Farwell et al., 2014 and error-related potentials, Chavarriaga et al., 2014) and the frequency domain (as workload index, Mühl et al., 2014 or in both, Hogervorst et al., 2014). Finally, Gerjets et al. (2014) and Zander and Jatzev (2012) argue that contextual information can be a useful addition to neurophysiological measures.

## Concluding remarks

The papers in this special issue show that the field of applied (neuro-) physiology is progressing in many aspects, especially in EEG-based passive Brain-Computer Interfaces. This is not to say that there are no remaining issues, but the field seems to be well-aware of the challenges it is facing (Gerjets et al., 2014; Strait and Scheutz, 2014; Brouwer et al., 2015) and the knowledge and technological breakthroughs it requires on the way to real-world applications. Also in order here is a word of caution: there are many potential pitfalls in using neurophysiological measures as listed by (Gerjets et al., 2014; Brouwer et al., 2015). However, and as also indicated, there are ways around them. The steps currently taken in applied neurophysiology also touch on other issues, such as ethics, acceptance of the technology by the general public, privacy of users, and the possible effects that these kinds of applications may have on society as a whole. These are not covered in this issue, but should also be borne in mind (Van Erp et al., 2012).

### Conflict of interest statement

The authors declare that the research was conducted in the absence of any commercial or financial relationships that could be construed as a potential conflict of interest.
